# Risk Factors for Moral Injury Among Canadian Armed Forces Personnel

**DOI:** 10.3389/fpsyt.2022.892320

**Published:** 2022-05-11

**Authors:** Bethany Easterbrook, Rachel A. Plouffe, Stephanie A. Houle, Aihua Liu, Margaret C. McKinnon, Andrea R. Ashbaugh, Natalie Mota, Tracie O. Afifi, Murray W. Enns, J. Don Richardson, Anthony Nazarov

**Affiliations:** ^1^MacDonald Franklin Operational Stress Injury Research Centre, London, ON, Canada; ^2^Department of Psychology, Neuroscience and Behaviour, McMaster University, Hamilton, ON, Canada; ^3^Homewood Research Institute, Guelph, ON, Canada; ^4^Department of Psychiatry, Western University, London, ON, Canada; ^5^School of Psychology, University of Ottawa, Ottawa, ON, Canada; ^6^Douglas Mental Health University Institute, Montreal, QC, Canada; ^7^Department of Psychiatry and Behavioural Neurosciences, McMaster University, Hamilton, ON, Canada; ^8^Department of Clinical Health Psychology, University of Manitoba, Winnipeg, MB, Canada; ^9^Department of Psychiatry, University of Manitoba, Winnipeg, MB, Canada; ^10^Department of Community Health Sciences, University of Manitoba, Winnipeg, MB, Canada; ^11^Deer Lodge Centre Operational Stress Injury Clinic, Winnipeg, MB, Canada

**Keywords:** mental health, deployment, military personnel, stress disorder, post-traumatic, moral injury, child maltreatment

## Abstract

**Objectives:**

The traumatic nature of high-risk military deployment events, such as combat, is well-recognized. However, whether other service-related events and demographic factors increase the risk of moral injury (MI), which is defined by consequences of highly stressful and morally-laden experiences, is poorly understood. Therefore, the objective of this study was to examine determinants of MI in Canadian Armed Forces (CAF) personnel.

**Methods:**

Data were obtained from the 2018 Canadian Armed Forces Members and Veterans Mental Health Follow-up Survey (CAFVMHS; unweighted *n* = 2,941). To identify military characteristics, sociodemographic variables, and deployment-related factors associated with increased levels of MI, a series of multiple linear regressions were conducted across deployed and non-deployed groups.

**Results:**

When all variables were considered among the deployed personnel, rank, experiencing military related sexual trauma, child maltreatment (i.e., physical abuse, emotional abuse and neglect), and stressful deployment experiences were significant predictors of increased MI total scores (β = 0.001 to β = 0.51, *p* < 0.05). Feeling responsible for the death of an ally and inability to respond in a threatening situation were the strongest predictors of MI among stressful deployment experiences. Within the non-deployed sample, experiencing military-related or civilian sexual trauma and rank were significant predictors of increased MI total scores (β = 0.02 to β = 0.81, *p* < 0.05).

**Conclusion:**

Exposure to stressful deployment experiences, particularly those involving moral-ethical challenges, sexual trauma, and childhood maltreatment were found to increase levels of MI in CAF personnel. These findings suggest several avenues of intervention, including education and policies aimed at mitigating sexual misconduct, as well as pre-deployment training to better prepare military personnel to deal effectively with morally injurious experiences.

## Introduction

Military service has been associated with an elevated risk of negative mental health outcomes including posttraumatic stress disorder (PTSD), depression, substance use, and suicidal behaviors globally (1–5). This finding holds in the Canadian context, with higher prevalence of mental disorders observed in Canadian Armed Forces (CAF) personnel compared to civilian populations (6, 7), with 44% of surveyed CAF members experiencing symptoms consistent with a depressive or anxiety- related disorder at some point between 2002 and 2018 (8).

Although stressful deployment experiences such as combat have been associated with increased negative mental health outcomes in military populations (1, 9), combat experiences are not the sole type of psychologically traumatic events military members may encounter. Exposure to stressful or difficult events with moral-ethical implications is also common (10–12), but the psychological distress associated with these experiences is less well understood. Therefore, it is critical to understand the pre-, peri- and post-deployment, as well as non-deployment experiences that are associated with moral injury in the CAF.

Moral injury (MI) refers to the psychological, spiritual, behavioral or social distress that follows from situations in which individuals have committed, witnessed, or failed to prevent acts that transgress one's personal moral beliefs (13, 14). These feelings of distress may include shame, guilt, anger, and disgust, which may be associated with acts perpetrated by the self, such as actions leading to loss of life, or acts perpetrated by others, including betrayal, witnessing inappropriate acts by colleagues, or inappropriate acts by individuals in positions of power (10–15). Morally injurious experiences, such as betrayal from a trusted peer, may prompt a variety of psychological, social, and behavioral consequences, including relational strain, fundamental shifts in core beliefs (e.g., beliefs about the world), spiritual/existential challenges, alterations in perceptions of the self, as well as feelings of guilt, shame or anger (10, 15, 16). Although evidence is currently limited, recent research indicates that potentially morally injurious experiences (PMIEs) are common, and may have a unique impact on post-deployment outcomes in military populations. A representative survey of United States (U.S.) military combat veterans found that approximately 25% of respondents reported witnessing transgressions of others, 25% reported experiencing betrayal during their careers, and 10% reported that they transgressed their personal morals (15). In a representative survey of CAF members deployed to the mission in Afghanistan, Nazarov et al. (11) found that over half of the population indicated experiencing at least one PMIE. The authors found that individuals indicating exposure to PMIEs were more likely to report experiencing past-year major depressive disorder (MDD) and past-year posttraumatic stress disorder (PTSD) while adjusting for other relevant variables such as age, sex, and deployment-related factors (11).

Although these findings provide evidence that certain PMIEs may increase the risk of negative mental health outcomes in deployed military members, there are specific limitations to the current body of research examining MI among military personnel. In the aforementioned study by Nazarov et al. (11), MI was not directly assessed using a validated measure; rather, mental health outcomes were assessed in relation to proxy deployment experiences used to indicate exposure to PMIEs (11). Wisco et al. (15) used the Moral Injury Events Scale (MIES) to assess MI, but because this study was conducted in a U.S. combat sample, the results may not generalize to the CAF due to cultural and structural differences between the two Armed Forces (15). Additionally, although both studies examined the impact that deployment PMIEs had on the development of other mental health disorders, the authors did not focus on factors that may increase the risk of development of MI among non-deployed personnel. Although this evidence suggests that PMIEs occur frequently during military combat and deployment operations, scant evidence exists regarding factors that may contribute to the development of MI in non-deployed CAF personnel. Understanding risk factors that contribute to the development of MI within both deployed and non-deployed CAF personnel is critical to appropriately target resilience-building interventions to mitigate development of MI.

### Aims of the Study

The aim of this study was to identify the military, deployment, and sociodemographic factors that are associated with increased MI in a nationally representative sample of CAF personnel and veterans. We hypothesized that deployment experiences and childhood maltreatment variables will significantly predict elevated MI scores in CAF personnel.

## Materials and Methods

### Participants and Data Collection

Data were obtained from the 2018 Canadian Armed Forces Members and Veterans Mental Health Follow-up Survey (CAFVMHS)(17). The CAFVMHS used a longitudinal survey design to resample individuals who initially participated in the 2002 Canadian Community Mental Health Survey—Mental Health and Well-being—Canadian Forces (CCHS-CF) (9). 5,155 CAF Regular Force personnel participated in the CCHS-CF in 2002, and 4,299 individuals were eligible to be contacted for follow-up interview. The target sampling frame for CAFVMHS were individuals who had completed the CCHS-CF and were full-time Regular Force members at the time of 2002 administration. At the time of 2018 data collection, personnel could be actively serving or veterans.

Of those who participated in the 2002 CCHS-CF and were eligible for follow-up (n = 4,299), 2,941 individuals participated in the CAFVMHS. Longitudinal weights were then created to produce representative estimates of the target population in 2002 and rounded to the nearest base of twenty. Therefore, the weighted survey sample represents 18,120 active duty and 34,380 released CAF personnel from the 2002 survey. As our analyses aimed to determine independent risk factors for the development of MI, and morally injurious experiences may differ between deployed and non-deployed personnel, the data were split into two groups: ever deployed outside North America and never-deployed groups. Data collection was conducted by Statistics Canada between January and May of 2018 using computer-assisted personal interviews. Participation was voluntary, and all participants provided informed consent. All data were collected in accordance with Statistics Canada procedures and approved by relevant review boards. For more information regarding the CAFVMHS rationale and methodology, please refer to (17, 18).

### Measures

#### Moral Injury

MI was evaluated using the Moral Injury Events Scale (19), which uses a six-point Likert scale to assess event experiences. Participants were provided a series of nine statements (e.g., “I am troubled by having witnessed others' immoral acts”) and were asked to indicate their level of agreement (1 = *strongly disagree*, 6 = *strongly agree*). Of note, logic skipping, wherein a participant selecting *strongly disagree* for certain items automatically imputed *strongly disagree* for a subsequent item, was used during administration [for more information, please see (20)]. Mean MIES scores were calculated and used as an outcome variable in our models, with higher mean scores indicating increased endorsement of MI. Past research has shown that while it is not without limitations (20), the MIES has strong evidence for internal consistency reliability and convergent validity (19, 20).

#### Deployment Experiences

Deployment experiences (DEX) were captured using a survey module that evaluated lifetime exposure and exposure since 2002 to eight stressful deployment experiences using dichotomous (*yes/no*) scoring (e.g., “known someone seriously injured or killed”). These items were adapted by the Canadian Department of National Defense (DND) from the Combat Experiences Scale (21). The eight items were chosen by the initial survey developers from the original Combat Experiences Scale instrument based on conceptual considerations (11).

#### Child Maltreatment

Participants were asked to retrospectively recall types of childhood adversity that they had been exposed to before the age of sixteen. Childhood physical abuse, sexual abuse, emotional abuse, exposure to intimate partner violence, and neglect were captured using nine items that were adapted from the Childhood Experiences of Violence Questionnaire (22). This measure has been used previously in population-level research to assess degree/severity of exposure to childhood adversity (11, 23). Of note, childhood sexual trauma was removed from the multivariate models due it theoretically being captured as a sub-category of lifetime sexual trauma.

#### Lifetime Sexual Trauma

Participants were asked if they had ever experienced sexual trauma in their lifetime. Sexual trauma was endorsed if they answered *yes* to one or more of eight dichotomous questions (e.g., “unwanted touching”). Further questions probed whether the event occurred while at a CAF workplace, while on deployment, or whether it was perpetrated by a CAF member/DND employee (17). If the respondent answered *yes* to any of these questions, these events were coded as military-related sexual trauma. If not, they were coded as non-military-related sexual trauma.

#### Military Variables

Previous research has shown that certain military variables may be associated with the presence of MI (11). As such, military variables, including force type, service environment (Army, Navy or Air Force), rank (junior non-commissioned member, senior non-commissioned officer, junior officer, senior officer), and number of years in the military, were included as covariates in our analyses (17). A dichotomous deployment variable was used to split the sample into CAF members who had deployed outside of North America and those who had not previously deployed. Separate models were created for deployed and non-deployed samples to independently assess how deployment-related variables impacted the endorsement of MI.

#### Demographic Covariates

Based on previous research that has shown associations between certain sociodemographic factors and MI, we adjusted for marital status, age, sex, and highest level of completed education in our analyses (11, 15). These variables were measured by self-report.

### Statistical Methods

First, we evaluated descriptive statistics across both samples, as well as simple linear regressions with MIES score as the outcome variable. Next, multiple linear regression models were conducted to assess military, deployment, and sociodemographic-related predictors of MI scores. Survey sample weights calculated by Statistics Canada were used in all analyses to ensure survey sample representativeness. Furthermore, to account for the complex survey design, confidence intervals were calculated using 500 bootstrapped weights provided by Statistics Canada. Based on Statistics Canada's vetting rules, reported frequencies used sample weights and were rounded on a base of twenty, with percentages calculated based on the weighted frequencies following rounding. Statistical analyses were conducted using SAS Version 9.4 (SAS Institute Inc., Cary, NC, USA).

## Results

The unweighted sample of 2,941 total participants represented 18,120 active duty and 34,380 released CAF personnel from the original 2002 survey. Over 90% (*n* = 39,600) of the deployed sample and 74% (*n* = 6,500) of the non-deployed sample were male. The majority of the deployed (69%, *n* = 30,300) and non-deployed (62%, *n* = 5,500) personnel were between the ages of 45–60 years at the time of the 2018 survey administration. Among those who deployed, stressful deployment experiences were commonly reported. Specifically, 62% endorsed knowing someone who had been seriously injured or killed, 46% had ever received incoming artillery, rocket or mortar fire, and 44% reported seeing injured or ill women or children they were unable to help (Table 1). Simple linear regressions with MIES total score as the outcome variable among deployed and non-deployed samples are displayed in Tables 2, 3, respectively. Force element (i.e., Army, Navy or Air Force) was a statistically significant predictor of MIES score in the deployed sample, though not in the non-deployed sample. Rank was a statistically significant predictor in both deployed and non-deployed samples.

**Table 1:** Sociodemographic and military characteristics of weighted study sample.

	**Deployed**		**Never deployed**	
	***n***	**Mean/percentage (95%CI)**	***n***	**Mean/percentage (95%CI)**
**Age**
33–44	9,240	21.14% (19.15–23.13%)	1,800	20.45% (16.27–24.64%)
45–60	30,300	69.34% (67.21–71.46%)	5,500	62.50% (58.02–66.98%)
61–75	4,160	9.52% (8.52–10.52%)	1,500	17.05% (14.25–19.84%)
**Sex**
Male	39,600	90.66% (90.14–91.18%)	6,500	73.86% (71.09–76.64%)
Female	4,080	9.34% (8.82–9.86%)	2,300	26.14% (23.36–28.91%)
**Education**
Secondary or lower	19,480	44.82% (42.50–47.14%)	2,900	32.95% (28.75–37.16%)
Postsecondary or higher	23,980	55.18% (52.86–57.50%)	5,900	67.05% (62.84–71.25%)
**Marital status**
Married	30,080	69.18% (67.23–71.13%)	5,900	67.05% (62.69–71.40%)
Common law	6,240	14.35% (12.77–15.93%)	1,040	11.82% (8.85–14.78%)
Separated/widowed/divorced	4,460	10.26% (8.91–11.60%)	1,280	14.55% (11.38–17.71%)
Single	2,700	6.21% (5.11–7.31%)	580	6.59% (4.36–8.83%)
**Military factors**
**Force type^†^**
Regular	38,760	88.82% (87.60–90.04%)	7,200	81.63% (78.45–84.81%)
Reserve	4,880	11.18% (9.96–12.40%)	1,620	18.37% (15.19–21.55%)
**Rank^†^**
Junior NCM	11,620	26.61%, (24.53–28.70%)	3,120	35.54%, (31.10–39.97%)
Senior NCO	22,160	50.76%, (48.68–52.83%)	2,900	33.03%, (29.08–36.98%)
Junior officer	3,200	7.33%, (6.31–8.35%)	1,020	11.62%, (9.43–13.81%)
Senior officer	6,680	15.30%, (14.31–16.29%)	1,740	19.82%, (17.04–22.59%)
**Service Environment**
Air Force	12,420	28.46% (26.55–30.38%)	4,820	54.77% (50.32–59.23%)
Army	23,020	52.75% (50.54–54.96%)	2,540	28.86% (24.59–33.14%)
Navy	8,200	18.79% (16.98–20.60%)	1,440	16.36% (13.09–19.64%)
**Years in military (mean)**		25.98 (25.68–26.28)		24.64 (23.73–25.56)
**Sexual trauma**
Place/person
No trauma	35,420	81.61% (79.94–83.28%)	6,620	75.92% (72.49–79.35%)
Military related^‡^	3,980	9.17% (8.03–10.31%)	1,020	11.70% (9.54–13.86%)
At other place or by others	4,000	9.22% (7.94–10.50%)	1,080	12.39% (9.57–15.21%)
**Child Maltreatment**
Physical abuse	19,640	45.17% (42.85–47.49%)	3,460	39.41% (34.95–43.87%)
Sexual	4,960	11.43% (10.02–12.84%)	1,080	12.30% (9.86–14.74%)
Exposure to intimate partner violence	5,320	12.21% (10.67–13.76%)	900	10.25% (7.62–12.88%)
Emotional abuse	8,400	19.41% (17.61–21.21%)	1,540	17.58% (14.53–20.63%)
Neglect	14,880	34.56% (32.37–36.74%)	2,300	26.38% (22.55–30.21%)
**Deployment experience**
Known someone seriously injured or killed	27,060	62.18% (59.92–64.43%)	-	-
In threatening situation—unable to respond due to rules of engagement	15,000	34.48% (32.23–36.73%)	-	-
Ever been injured	15,300	35.19% (33.05–37.33%)	-	-
Ever received incoming artillery, rocket or mortar fire	20,000	46.00% (43.72–48.28%)	-	-
Had close call, e.g. shot/hit but were protected	11,100	25.54% (23.47–27.61%)	-	-
Seen ill/injured women/children who you were unable to help	19,140	44.04% (41.72–46.36%)	-	-
Felt responsible for death of Canadian or ally personnel	3,220	7.41% (6.14–8.68%)	-	-
Difficulty distinguishing between combatants and non-combatants	13,620	31.31% (29.12–33.5%)	-	-

† *Force type and Rank in 2018*.

*NCM, non-commissioned member; NCO, non-commissioned officer*.

‡ *Military-related: occurred at CAF workplace or perpetrated by CAF member/DND staff*.

**Table 2:** Simple linear regressions predicting MIES scores among deployed CAF personnel (weighted *n* = 43,700).

**Variables**	**Standardized regression**	**Standard error**	***t*-value**	***p*-value**	***R*** **^2^**
	**coefficient**
**Age**					0.0020
33–44 (ref)
45–60	0.11	0.061	1.86	0.0628	
61–75	−0.0046	0.096	−0.05	0.9624	
**Sex**					0.0026
Male (ref)
Female	0.21	0.085	2.47	0.0137	
**Education**					0.0029
Secondary or lower (ref)
Postsecondary or higher	−0.13	0.05	−2.57	0.0104	
**Marital status**					0.0092
Married (ref)
Common law	0.22	0.072	3.05	0.0023	
Separated/widowed/divorced	0.32	0.083	3.84	0.0001	
Single	0.12	0.104	1.13	0.2568	
**Military factors**
**Force type^†^**					0.0024
Regular (ref)
Reserve	−0.18	0.078	−2.34	0.0196	
**Service Environment**					0.0096
Army (ref)
Air Force	−0.24	0.057	−4.18	<0.0001	
Navy	−0.22	0.066	−3.38	0.0007	
**Rank^†^**					0.0276
Junior NCM	0.62	0.078	7.96	<0.0001	
Senior NCO	0.41	0.071	5.82	<0.0001	
Junior officer	0.28	0.11	2.53	0.0115	
Senior officer (ref)
**Years in military**	−0.0083	0.0034	−2.46	0.0140	0.0026
**Sexual trauma**					
Place/person					0.0436
No trauma (ref)
Military related^‡^	0.86	0.089	9.69	<0.0001	
At other place or by others	0.34	0.081	4.23	<0.0001	
Relate to deployment or not					0.0440
No trauma (ref)
While deployment	1.03	0.115	9.01	<0.0001	
Not while deployment	0.40	0.071	5.72	<0.0001	
**Type of sexual trauma**
Sexual assault					0.0413
No trauma (ref)					
Military related^‡^	1.11	0.12	9.22	<0.0001	
Non-military	0.47	0.11	4.19	<0.0001	
Sexual unwanted touching					0.0434
No trauma (ref)
Military related^‡^	0.86	0.09	9.71	<0.0001	
Non-military	0.33	0.08	3.92	<0.0001	
**Sexual assault or unwanted touching**
No trauma (ref)					0.0482
Military related^‡^	0.89	0.08	10.60	<0.0001	
Non-military	0.25	0.08	3.01	0.0027	
**Child Maltreatment**					
Physical	0.48	0.049	9.85	<0.0001	0.0405
Sexual	0.52	0.077	6.76	<0.0001	0.0195
Exposure to intimate partner violence	0.39	0.075	5.24	<0.0001	0.0118
Emotional abuse	0.78	0.061	12.95	<0.0001	0.0681
Neglect	0.46	0.051	8.90	<0.0001	0.0335
**Deployment experience**					
Known someone seriously injured or killed	0.51	0.050	10.20	<0.0001	0.0432
In threatening situation—unable to respond due to rules of engagement	0.66	0.050	13.14	<0.0001	0.0697
Ever been injured	0.55	0.050	10.88	<0.0001	0.0489
Ever received incoming artillery, rocket or mortar fire	0.25	0.049	5.15	<0.0001	0.0114
Had close call, e.g., shot/hit but were protected	0.57	0.055	10.30	<0.0001	0.0441
Seen ill/injured women/children who you were unable to help	0.62	0.048	12.83	<0.0001	0.0667
Felt responsible for death of Canadian or ally personnel	0.88	0.092	9.53	<0.0001	0.0380
Difficulty distinguishing between combatants and non-combatants	0.52	0.052	9.95	<0.0001	0.0412

† *Force type and Rank in 2018*.

‡ *Military related is defined as the sexual trauma that happened in CAF workplace or by CAF member/DND staff or while on deployment*.

*NCO, non-commissioned officer; NCM, non-commissioned member*.

**Table 3:** Simple linear regressions predicting MIES scores among non-deployed CAF personnel (weighted *n* = 8,800).

**Variables**	**Standardized regression coefficient**	**Standard error**	***t*-value**	***p*-value**	***R*** **^2^**
**Age**					0.0073
33–44 (ref)
45–60	0.16	0.12	1.41	0.1588	
61–75	−0.071	0.15	−0.48	0.6343	
**Sex**					0.0309
Male (ref)
Female	0.45	0.10	4.46	<0.0001	
**Education**					0
Secondary or lower (ref)
Postsecondary or higher	0.012	0.096	0.12	0.9008	
**Marital status**					0.0156
Married (ref)
Common law	0.15	0.14	1.04	0.2991	
Separated/widowed/divorced	0.40	0.13	3.08	0.0021	
Single	0.050	0.18	0.26	0.7986	
**Military factors**
**Force type^†^**					0.0008
Regular (ref)
Reserve	−0.085	0.12	−00.73	0.4680	
**Service Environment**					0.0047
Army (ref)
Air Force	−0.15	0.10	−1.44	0.1517	
Navy	−0.21	0.14	−1.53	0.1270	
**Rank^†^**					0.0366
Junior NCM	0.58	0.12	4.67	<0.0001	
Senior NCO	0.25	0.13	1.94	0.0533	
Junior officer	0.32	0.16	1.93	0.0537	
Senior officer (ref)
**Years in military**	0.00027	0.0047	0.06	0.9542	0
**Sexual trauma**					
Place/person					0.1035
No trauma (ref)
Military related	0.99	0.14	7.31	<0.0001	
At other place or by others	0.68	0.13	5.15	<0.0001	
**Type of sexual trauma**
**Sexual assault**					0.095
No trauma (ref)					
Military related^‡^	1.20	0.18	6.79	<0.0001	
Non-military	0.82	0.17	4.80	<0.0001	
**Sexual unwanted touching**					0.1028
No trauma (ref)
Military related^‡^	1.02	0.14	7.38	<0.0001	
Non-military	0.66	0.14	4.81	<0.0001	
**Sexual assault or unwanted touching**					0.1035
No trauma (ref)
Military related^‡^	0.99	0.14	7.31	<0.0001	
Non-military	0.68	0.13	5.15	<0.0001	
**Child Maltreatment**
Physical	0.34	0.09	3.73	0.0002	0.0219
Sexual	0.78	0.13	5.79	<0.0001	0.0512
Exposure to intimate partner violence	0.37	0.15	2.51	0.0125	0.010
Emotional abuse	0.58	0.12	4.99	<0.0001	0.0385
Neglect	0.35	0.10	3.38	0.0008	0.0182

† *Force type in 2018*.

‡ *Military related is defined as the sexual trauma that happened in CAF workplace or by CAF/DND staff or while on deployment*.

*NCM, non-commissioned member; NCO, non-commissioned officer*.

Multiple linear regression models to determine independent risk factors for increased MI score are reported in Tables 4, 5. The independent variables accounted for approximately 25% of the variance in MI scores in the deployed sample and 17% in non-deployed CAF personnel. Rank, years in military, military-related sexual trauma, childhood physical and emotional abuse, childhood neglect, and stressful deployment experiences were predictors of increased MI score in the deployed sample (Table 4). When all variables were included in the model, the strongest deployment-related predictors of higher MI score were feeling responsible for the death of an ally and inability to respond in a threatening situation due to rules of engagement. Within the non-deployed sample, rank, experiencing sexual trauma (military or civilian), years in the military, and childhood neglect were the only significant predictors of increased MI scores (Table 5).

**Table 4:** Multiple linear regression model of MIES scores regressed on military/sociodemographic factors among deployed CAF personnel (weighted *n* = 43,700).

**Variables**	**Standardized regression coefficient**	**Standard error**	***t*-value**	***p*-value**
**Demographics**
**Sex**
Male (ref)
Female	0.14	0.09	1.62	0.1059
**Education**
Secondary or lower (ref)
Postsecondary or higher	−0.02	0.05	−0.40	0.6868
**Military factors**
**Force type^†^**
Regular (ref)
Reserve	−0.04	0.07	−0.62	0.5373
**Rank^†^**
Junior NCM	0.39	0.08	4.91	<0.0001
Senior NCO	0.26	0.07	3.70	0.0002
Junior officer	0.16	0.10	1.65	0.0994
Senior officer (ref)
**Years in military**	0.001	0.003	2.66	0.0078
**Sexual assault or unwanted sexual touching**
No trauma (ref)
Military-related^‡^	0.61	0.09	6.98	<0.0001
Non-military	0.10	0.08	1.33	0.1831
**Child maltreatment**
Physical abuse	0.19	0.05	3.73	0.0002
Exposure to intimate partner violence	−0.04	0.07	−00.50	0.6194
Emotional abuse	0.48	0.06	7.39	<0.0001
Neglect	0.19	0.05	3.75	0.0002
**Deployment experience**
Known someone seriously injured or killed	0.09	0.05	1.75	0.0809
In threatening situation—unable to resp. bc of rules of engage	0.27	0.06	4.86	<0.0001
Ever been injured	0.19	0.05	3.68	0.0002
Ever received incoming artillery, rocket or mortar fire	−0.13	0.05	−2.49	0.0127
Had close call, e.g., shot/hit but were protected	0.22	0.06	3.62	0.0003
Seen ill/injured women/children who you were unable to help	0.19	0.05	3.56	0.0004
Felt responsible for death of Canadian or ally personnel	0.51	0.09	5.72	<0.0001
Difficulty distinguishing between combatants and non-combatants	0.19	0.06	3.31	0.0009

† *Force type and Rank in 2018*.

‡ *Military related is defined as the sexual trauma that happened in CAF workplace or by CAF/DND staff or while on deployment*.

*NCM, non-commissioned member; NCO, non-commissioned officer*.

**Table 5:** Multiple linear regression model of MIES scores regressed on military/sociodemographic factors among non-deployed CAF personnel (weighted *n* = 8,800).

**Variables**	**Standardized regression coefficient**	**Standard error**	***t*-value**	***p*-value**
**Demographics**
**Sex**
Male (ref)
Female	0.10	0.12	0.85	0.3955
**Education**
Secondary or lower (ref)				
Postsecondary or higher	0.18	0.10	1.88	0.0604
**Military factors**
**Force type^†^**
Regular (ref)
Reserve	−0.12	0.11	−1.09	0.2760
**Rank^†^**
Junior NCM	0.80	0.14	5.63	<0.0001
Senior NCO	0.33	0.13	2.56	0.0108
Junior Officer	0.31	0.16	1.93	0.0544
Senior officer (ref)
**Years in military**	0.02	0.001	4.00	<0.0001
**Sexual assault or unwanted sexual touching**
No trauma (ref)
Military-related	0.81	0.16	5.13	<0.0001
Non-military	0.54	0.14	3.84	0.0001
**Child maltreatment**
Physical	0.12	0.10	1.24	0.2139
Exposure to intimate partner violence	−0.04	0.16	−0.23	0.8189
Emotional abuse	0.14	0.14	1.06	0.2902
Neglect	0.22	0.11	2.09	0.0371

† *Force type and Rank in 2018*.

*NCM, non-commissioned member; NCO, non-commissioned officer*.

## Discussion

This is the first study to identify factors associated with increased MI using a representative survey of Canadian military personnel. Among non-deployed CAF personnel, experiencing either military-related or civilian sexual trauma, and junior non-commissioned member rank (compared to senior officer) were significantly associated with increased MI total scores. Among the previously deployed CAF personnel, child maltreatment (i.e., neglect, physical abuse and emotional abuse), experiencing military-related sexual trauma, and stressful deployment experiences (e.g., feeling responsible for the death of an ally) were significant predictors of MI total scores.

Specific military variables, including deployment experiences and individual rank, were independently associated with MIES score in deployed personnel. These experiences, such as seeing ill or injured children and being unable to help, may be categorized as PMIEs as they are situations that may lead to the violation of moral values (24), a precursor to MI. Further, in both deployed and non-deployed samples, rank was independently associated with MIES score, which is consistent with previous findings (11). Interestingly with regards to rank, being a junior non-commissioned member, regardless of deployment status, conferred the strongest association with MIES scores when compared to senior officers. This could be due to a multitude of factors, including differences in duties, increased likelihood of deployment related PMIEs, and power structure dynamics inherent in the military rank system.

Importantly, sexual trauma was a significant predictor of MIES score in the simple linear regression models for both deployed and non-deployed CAF members, perhaps due to feelings of perceived betrayal from these experiences (25). However, when all variables were considered together, military sexual trauma was the only sexual trauma variable significantly associated with MIES score in deployed CAF personnel. Military sexual trauma perpetrated by CAF personnel or DND staff or at a CAF workplace, defined in this study as unwanted touching or sexual assault, was a significant predictor of increased MIES score in both the deployed and non-deployed samples. These definitions largely overlap with the concept of Military Sexual Misconduct (MSM), which has been associated with adverse mental and physical health outcomes, including PTSD, in U.S. military populations (26, 27). In 2018, 70% of CAF respondents reported experiencing targeted MSM during the previous 12 months of military service (28), indicating that this is a pervasive and preventable risk factor for the development of MI. Although civilian sexual trauma was not a significant predictor of MI in deployed CAF personnel, it did significantly predict MI scores in the non-deployed sample and among both simple linear regression models. It is plausible that there was overlapping variance between, for example, civilian sexual trauma and other variables (e.g., childhood maltreatment) that rendered these associations non-significant in the full deployed model. Additional research regarding the relative risk of civilian and military-related sexual trauma and their overlap in both deployed and non-deployed samples is warranted. Such studies are likely to shed additional light on the mechanisms and contextual factors associated with the development of MI.

Our analyses further indicated that childhood physical and emotional abuse and childhood neglect were positive predictors of increased MI scores in deployed CAF personnel, though only childhood neglect was a positive predictor in non-deployed personnel. The deployed sample results were consistent with previous findings in treatment-seeking CAF Veteran convenience samples (29). Consistent with our findings, a history of childhood abuse and its implications for negative mental and physical health outcomes in adults has been well-documented (30–35). In the same way that research has shown that childhood/earlier traumatic experiences increase risk for exposure to future trauma and PTSD (23), these findings indicate that the same may be true for PMIEs and MI, with increased exposure to PMIEs in childhood possibly increasing the risk for exposure to other PMIEs or development of MI later in life. Although childhood trauma variables except neglect were not significant predictors of increased MI in non-deployed personnel, there were significant associations between childhood maltreatment variables and MIES scores in the simple regression models. It is plausible, then, that child maltreatment shared common variance with non-military-related sexual trauma that attenuated the associations between childhood maltreatment variables and MIES scores.

### Limitations

Although the findings of this study provide novel information regarding predictors of MI in deployed and non-deployed CAF personnel, we acknowledge several limitations. Due to the longitudinal nature of the CAFVMHS, the 2018 sample is representative of the original 2002 CAF sample that took part in the initial survey and is not necessarily representative of current CAF demographics. In addition, because the sample was primarily composed of men, this limited our ability to assess how sex and gender may be associated with moral distress in the CAF. Furthermore, variables included in the analyses are not an exhaustive list of potential predictors of MI, especially given that the study of MI remains in its infancy. Importantly, psychological traumas external to military experiences aside from sexual assault were not included in analysis, as the MIES alludes exclusively to military experiences. There is also the possibility that other peri-deployment or post-deployment experiences captured in this survey that were not included in the analyses may have influenced the endorsement of MI. Due to response bias, there may also be unknown differences between survey responders and non-responders, which may theoretically have altered findings of this study. However, previous research on attrition in this sample found that military status, mental health disorders, traumatic experiences and childhood adversity were not associated with loss to follow-up (18).

Childhood maltreatment was also assessed retrospectively during adulthood in this survey, which may introduce recall bias. However, research indicates that this is unlikely, as retrospective recall of childhood trauma seems to be reliable (18, 36, 37). Although relevant literature points to a strong correlation between childhood sexual abuse and negative mental health outcomes (38–43), childhood sexual trauma was not included in the regression models due to being captured by the item endorsing lifetime sexual trauma. This precluded us from determining how or whether childhood sexual trauma may influence MI endorsement in this population.

Although it is currently the most widely used measure of MI, the MIES has been previously criticized for conflating MI exposure and subjective experience without differentiating between the constructs during scoring, which may inadvertently introduce extraneous variance when attempting to determine severity of MI (20). The subjective self-report nature of the measure, as well as the logic skipping that was used during Statistics Canada administration may also have introduced response biases in the survey. The CAFVMHS 2018 MIES scoring logic, wherein a participant selecting *strongly disagree* for certain items automatically imputed *strongly disagree* for a subsequent item, could have created issues with total MIES scoring. However, following previous research (20) regarding MIES response patterns in this population, we believe that it is unlikely that this logic skipping introduced bias within the survey.

Future directions should include assessing MI using a scale that focuses on the expressed outcomes that make up the MI construct (e.g., spiritual struggles, guilt) and investigate the nuances present in how exposures and outcomes are related. Since the time that data were collected for this study, a number of measures that clearly differentiate outcomes of PMIEs from exposures to PMIEs have been developed, although additional psychometric validation for these measures is warranted. Future research should also consider separate risk factors for endorsement of MI that were not captured in this survey, such as personality traits. Finally, while consensus is amounting that MI is a clinically useful construct [e.g., (44, 45)], additional research is needed to establish effective screening and intervention strategies within military and other populations at heightened risk of MI. Implications of these results indicate that specific care should be taken to incorporate discussion surrounding MI, and tailored treatments to reduce symptoms of MI (e.g., anger, shame) within treatment-seeking military contexts. Focus of future interventions should also be placed on pre-deployment training and preparation for military personnel to effectively understand and cope with morally injurious experiences.

Notwithstanding these limitations, this is the first study to evaluate predictors of MI endorsement in a representative sample of CAF personnel. Our findings emphasize the critical importance of explicitly screening for and addressing deployment experiences and military sexual trauma in the context of evaluating and addressing MI in military populations. Results also point to several demographic and developmental factors that should be further investigated in future research aiming to understand individual vulnerability to MI.

## Data Availability Statement

The datasets presented in this article are not readily available because the data can be accessed in Canada with permission of Statistics Canada through the Statistics Canada Research Data Centers. Statistics Canada collected and provided the data for academic purposes, but the analyses are the sole responsibility of the authors. The opinions expressed do not represent the views of Statistics Canada. Requests to access the datasets should be directed to Statistics Canada's Statistical Information Service, STATCAN.infostats-infostats.STATCAN@canada.ca. Further enquiries can be directed to the corresponding author.

## Ethics Statement

The studies involving human participants were reviewed and approved by Statistics Canada. The patients/participants provided their written informed consent to participate in this study.

## Author Contributions

BE, AN, SH, RP, AL, MM, and JR contributed to the conception, design of this project, and made changes in online comments. BE, AN, SH, RP, and AL created the conceptual models. AL conducted the analyses. BE wrote the first draft of the manuscript. SH, RP, AA, NM, TA, and ME made important contributions to sections of the manuscript. All authors contributed to manuscript revision, read, and approved the submitted version.

## Conflict of Interest

The authors declare that the research was conducted in the absence of any commercial or financial relationships that could be construed as a potential conflict of interest.

## Publisher's Note

All claims expressed in this article are solely those of the authors and do not necessarily represent those of their affiliated organizations, or those of the publisher, the editors and the reviewers. Any product that may be evaluated in this article, or claim that may be made by its manufacturer, is not guaranteed or endorsed by the publisher.
